# Hypoxia Regulates the Expression of the Neuromedin B Receptor through a Mechanism Dependent on Hypoxia-Inducible Factor-1α

**DOI:** 10.1371/journal.pone.0082868

**Published:** 2013-12-09

**Authors:** Hyun-Joo Park, Mi-Kyoung Kim, Su-Ryun Kim, Soo-Kyung Bae, Moon-Kyoung Bae

**Affiliations:** 1 Department of Oral Physiology, School of Dentistry, Pusan National University, Yangsan, South Korea; 2 Department of Dental Pharmacology, School of Dentistry, Pusan National University, Yangsan, South Korea; Sun Yat-sen University Medical School, China

## Abstract

The neuromedin B receptor (NMB-R), a member of the mammalian bombesin receptor family, is frequently overexpressed in various tumors. In the present study, we found that exposure to hypoxic conditions increases the levels of *NMBR* mRNA and protein in breast cancer cells, which are tightly regulated by hypoxia-inducible factor-1α (HIF-1α). We confirmed the effect of HIF-1α on *NMBR* transcription by performing an *NMBR* promoter-driven reporter assay and then identified a functional hypoxia-responsive element (HRE) in the human *NMBR* promoter region. Further, the binding of HIF-1α to the *NMBR* promoter was corroborated by electrophoretic mobility shift and chromatin immunoprecipitation assays, which showed that HIF-1α specifically and directly bound to the *NMBR* promoter in response to hypoxia. Immunohistochemical analysis of a xenograft and a human breast cancer tissue array revealed a significant correlation between NMB-R and HIF-1α expression. Taken together, our findings indicate that hypoxia induces NMB-R expression through a novel mechanism to regulate HIF-1α expression in breast cancer cells.

## Introduction

Neuromedin B (NMB), a member of a family of mammalian bombesin-like peptides, exerts diverse physiological effects on a number of functions, including smooth-muscle contraction, exocrine and endocrine secretion, regulation of blood pressure and glucose levels, and normal cellular growth [1,2]. Bombesin-like peptides, including NMB and gastrin-releasing peptide (GRP), are released by malignant tumor cells and act as autocrine growth factors and mitogens that influence proliferation and cell cycle progression [3-5]. NMB plays an important role in stimulating tumor growth and tumor angiogenesis through its cognate receptor, NMB-R [6,7]. Inhibition of NMB-R activity efficiently suppresses the growth and colony-forming ability of breast cancer cells [8]. NMBR is aberrantly expressed by various types of solid tumors such as lung, prostate, colorectal, and breast tumors [8-10]. However, the mechanism that regulates *NMBR* expression in cancer cells exposed to environmental stimuli is unknown.

Low oxygen levels detected in the central area of solid tumors have been identified as an essential determinant of angiogenesis, which is required for the growth of malignant tumors [11,12]. A crucial transcription factor in angiogenesis is hypoxia-inducible factor (HIF), a heterodimer of HIF-1α and HIF-1β, that controls transcription of hypoxia-regulated genes encoding vascular endothelial growth factor, vascular endothelial growth factor receptor-1, glucose transporter 1, and stromal-derived factor-1 [13-15].

Here, we investigated the effect of hypoxia on the expression of NMB-R and determined whether HIF-1α directly influences hypoxic induction of NMB-R in MDA-MB-231 breast cancer cells. To the best of our knowledge, this study is the first to report that *NMBR* is hypoxia-responsive in breast cancer cells and to elucidate the mechanisms underlying its regulation.

## Materials and Methods

Ethical approval was given by the Institutional Animal Care and Use Committee at Pusan National University, Korea

### Reagents and antibodies

l-Mimosine, dimethyloxaloylglycine (DMOG), and YC-1 were purchased from Sigma Aldrich. Mouse monoclonal anti-hypoxia-inducible factor-1 (HIF-1α) and rabbit polyclonal anti-neuromedin B receptor (NMB-R) antibodies were obtained from Novus Biologicals and Santa Cruz Biotechnology, respectively. A human-specific anti-α-tubulin antibody was purchased from Biogenex. Horseradish peroxidase-conjugated goat anti-rabbit and anti-mouse IgG were purchased from Thermo Fisher Scientific. Alexa® 488-conjugated goat anti-mouse and Alexa® 594-conjugated goat anti-rabbit IgGs were purchased from Life Technologies.

### Cell culture and hypoxic conditions

MDA-MB-231, MDA-MB-468, and MCF-7 cell lines (American Type Culture Collection [ATCC]) were cultured in DMEM containing 10% heat-inactivated FBS and 1% antibiotics (all from Life Technologies) at 37 °C in a humidified atmosphere containing 5% CO_2_. T47D (ATCC) cells were maintained in RPMI-1640 media (Life Technologies) containing insulin (5 μg/ml), FBS (10%) and antibiotics (1%) at 37 °C in a humidified (5%) incubator. For hypoxic condition, cells were incubated in 5% CO_2_, and 1% O_2_ balanced with N_2_ in a hypoxic chamber.

### RT-PCR analysis

Total RNA was isolated from MDA-MB-231 cells with a TRIzol reagent kit (Life Technologies). cDNA synthesis was performed using 2 μg of total RNA with a reverse transcription kit (Promega). The oligonucleotide primers for PCR were as follows: *β-actin* (*ACTB*), 5′-GACTACCTCATGAAGATC-3′ and 5′-GATCCACATCTGCTGGAA-3′; *NMBR*, 5′-CAGAAGTGGCTCGCATCAGT-3′ and 5′-GCTGTTGAAATGCCTCCTGA-3′.

### Real-time PCR analysis

Real-time PCR quantification was performed using a SYBR® Green method (Light Cycler; Roche Applied Science). Cycling parameters included 1 cycle at 95 °C for 10 min, followed by amplification for 30 cycles at 95 °C for 10 s, 57 °C for 5 s, and 72 °C for 7 s. A melting curve program was subsequently applied with continuous measurements of fluorescence. The entire cycling process, including data analysis, took less than 1 h and was monitored using Light Cycler software (version 4.0). The oligonucleotide primers for real-time PCR were as follows: *ACTB*, 5′-ACTCTTCCAGCCTTCCTTCC-3′ and 5′-TGTTGGCGTACAGGTCTTTG-3′; *NMBR*, 5′-CAGAAGTGGCTCGCATCAGT-3′ and 5′-CAGGAAGATTGTGTGCGCTT-3′.

### Western blot analysis

Harvested cells were lysed in a buffer containing 40 mm Tris-Cl, 10 mm EDTA, 120 mm NaCl, 0.1% Nonidet P-40, and a protease inhibitor cocktail (Sigma Aldrich)]. Samples contained an equal amount of protein (30 μg/lane), were separated using SDS-PAGE, and transferred to a nitrocellulose membrane (GE Healthcare Life Sciences). The membrane was blocked with 5% skim milk in PBS containing 0.1% Tween 20 for 1 h at room temperature and probed with appropriate antibodies. The signal was developed using the enhanced chemiluminescence (ECL) detection system (GE Healthcare Life Sciences).

### Immunocytochemistry

Cells cultured on a coverglass were fixed in 4% paraformaldehyde for 10 min, blocked with 0.5% Triton X-100/PBS for 5 min, and then reacted with appropriate primary antibodies and Alexa® 488 and 594-conjugated secondary antibodies. Coverslips were mounted in Vectastain containing DAPI (Vector Laboratories). Cells were analyzed using fluorescence microscopy (Nikon).

### RNA interference

MDA-MB-231 cells were transfected with an siRNA specific for HIF-1α (antisense: 5´-UCAAACACACUGUGUCCAG-3´; sense: 5´-CUGGACACAGUGUGUUUGA-3´) (100 nM) using Oligofectamine™ (Life Technologies). After transfection, the cells were exposed to a normoxic or hypoxic atmosphere for the indicated times. Negative-control siRNA and *NMB*R siRNAs were purchased from Bioneer and Santa Cruz Biotechnology, respectively.

### Plasmids and constructs

A genomic DNA fragment of *NMB* (NCBI Reference Sequence: NT_025741.15) containing ~1.5 kb of 5'-flanking region was prepared by PCR amplification of human genomic DNA. A 1259-bp PCR product was obtained and subcloned into a pCR2.1/TA vector (Life Technologies). This construct was amplified using PCR and subcloned into the pGL3 luciferase reporter vector (Promega). All constructs were confirmed using automatic DNA sequencing analysis (Cosmo Genetech). A vector that expresses human *NMB* was obtained from OriGene.

### Transient transfection and reporter gene analysis

Cells were seeded onto 24-well plates and transfected with luciferase constructs and pCMV-β-gal using Lipofectamine® 2000 (Life Technologies). The next day, the cells were incubated under hypoxic conditions for 24 h. Cell lysates were analyzed for β-galactosidase and luciferase activities using an assay kit (Promega) and luminometer (Turner Designs). Luciferase activity, normalized to that of β-galactosidase, is expressed as the average of 3 independent experiments.

### BrdU incorporation assay

To evaluate cell proliferation, we used an FITC BrdU Flow kit (BD Biosciences) according to the manufacturer’s protocol. MDA-MB-231 cells were transfected with the *NMBR* expression vector or *NMBR* siRNA and then exposed to normoxic or hypoxic conditions. Cultured cells were then labeled with BrdU for 3 h, washed, fixed, and permeabilized with BD Cytofix/Cytoperm buffer. After repeated incubation on ice, washes, and centrifugation, cells were treated with DNase for 1 h at 37 °C to expose the BrdU epitope, washed, stained with fluorescent anti-BrdU antibody for 20 min at room temperature, washed again, and analyzed using a FACS Calibur (BD Bioscience).

### Site-directed mutagenesis

Three putative HIF-1 binding sites (HRE, 5'-RCGTG-3') within p(1259)luc were targeted for mutagenesis. The mutations were made using a Quick Change site-directed mutagenesis kit (Stratagene), and the sequences were confirmed using automatic DNA sequencing (Cosmo Genetech).

### Chromatin immunoprecipitation (ChIP) assay

ChIP analysis was performed with the ChIP assay kit (Millipore), according to the manufacturer’s protocol. Immunoprecipitation was performed with control IgG and anti-HIF-1α antibodies. The region containing HIF-1α–binding sites within the human *NMBR* promoter was amplified using PCR with specific primers. Individual ChIP assays were repeated 3 times.

### Electrophoretic mobility shift assay (EMSA)

Nuclear extracts from MDA-MB-231 cells were prepared and analyzed by EMSA. Binding reactions containing equal amounts of nuclear extracts (1 μg) and biotin-labeled oligonucleotide were performed for 20 min in binding buffer. The binding reactions were analyzed using 6% native PAGE. After blotting onto a nylon membrane, labeled oligonucleotides were detected with the Light Shift Chemiluminescent EMSA Kit following the instructions of the manufacturer (Thermo Fisher Scientific).

### Tumor xenograft and animal studies

MDA-MB-231 cells (1 × 10^7^ cells) were subcutaneously injected into 6 weeks of age female athymic nude mice. Mice were divided into 3 groups of 5 animals each when tumor size grew larger than 4 mm in diameter. To visualize the hypoxic region in the tumor tissue, 60 mg/kg of pimonidazole was intravenously administered 30–45 min before perfusion fixation. All animal care and experiments were performed in accordance with the Institutional Guidelines of the Animal Care and Use Committees of Pusan National University.

### Immunohistochemistry

Human breast cancer-tissue microarrays were purchased from Super Bio Chips (SuperBioChips Laboratories, Seoul, KOREA). The tumor tissues were obtained from surgical specimens of patients. No clinical information except the age and gender of each patient was available for the tissue on these arrays. Xenograft tissues were prepared, and thin sections (4 μm) from selected areas were used. In brief, after deparaffinization and blocking of endogenous peroxidase activity, antigen retrieval was routinely performed using PBS containing 0.04% alkaline protease solution (Promega). The primary antibodies used in this study were mouse anti-CD31, rabbit anti-NMB-R and mouse anti-HIF-1α. To detect these antibodies, Alexa Fluor® 488-conjugated anti-mouse IgG (1:200) and Alexa Fluor 594®-conjugated anti-rabbit IgG (1:200) were used. Isotype controls were stained with either one of the secondary antibodies to verify specificity. Immunofluorescence staining was performed and visualized using a Nikon digital sight DS-SMc camera attached to a Nikon ECLIPSE 55i microscope.

### Statistical analysis

Data are presented as the mean ± standard deviation (sd) obtained from at least 3 independent experiments. Statistical comparisons between groups were performed using one-way ANOVA followed by the Student’s *t*-test.

## Results

### Hypoxia increases expression of NMB-R mRNA and protein by human breast cancer cell lines

**Figure 1:**
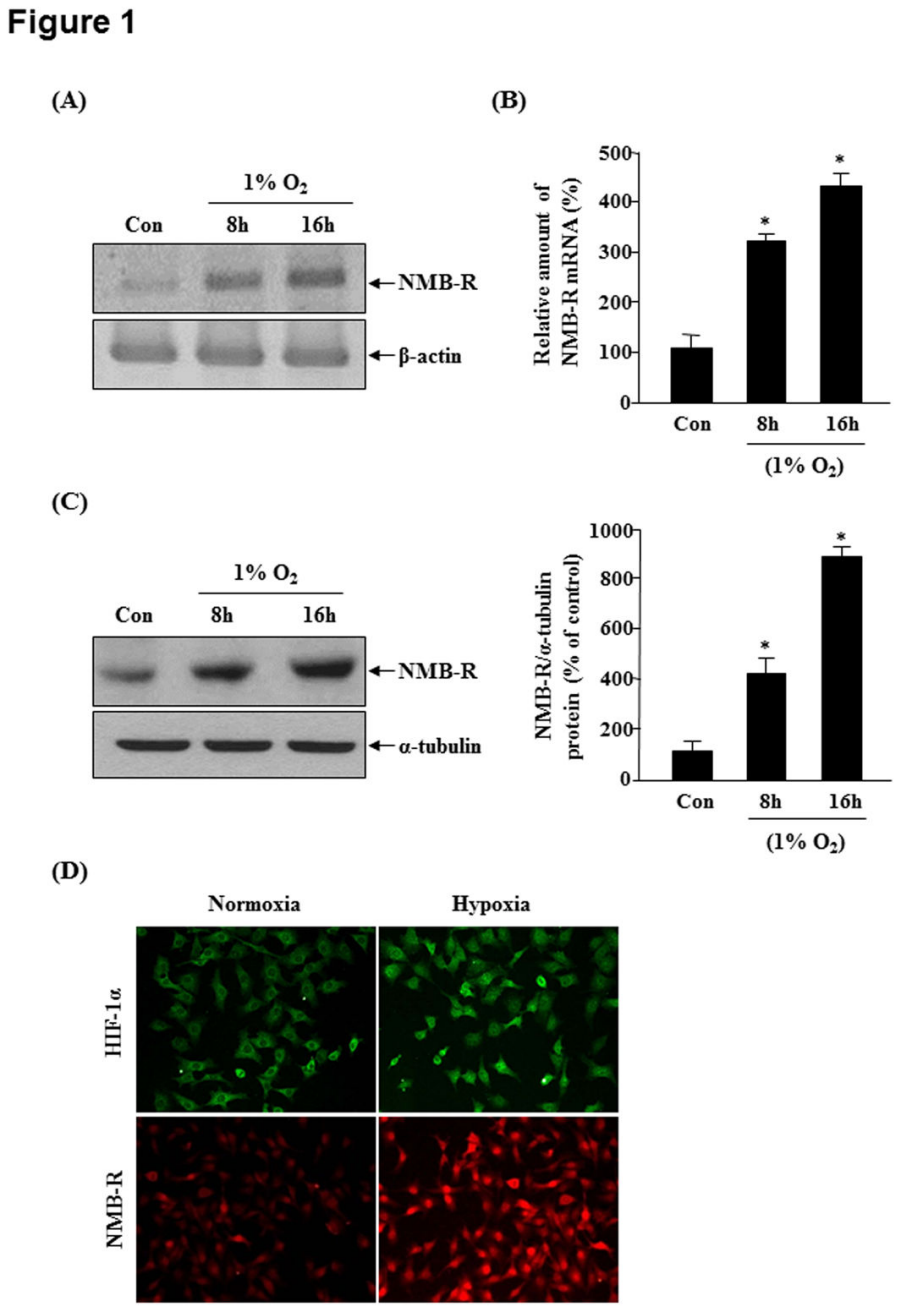
Effect of hypoxia on NMBR expression. MDA-MB-231 cells were incubated under normoxic (21% O_2_) or hypoxic (1% O_2_) conditions for the indicated times. (A) Total RNA was isolated and analyzed using RT-PCR with primers specific for human NMBR. β-actin was used as the internal control. (B) Using real-time PCR, the expression levels of NMBR mRNA were quantified. The expression level of the control (untreated) was defined as 100%, and the values were normalized to those of β-actin mRNA levels. (C) The expression of NMB-R was examined by western blotting using an anti-NMB-R antibody, and α-tubulin served as the loading control (left). The right panel shows the densitometric analysis of relative NMB-R expression levels in at least 3 independent experiments. *P<0.05 vs. control. (D) Higher magnification images showed immunoreactivity of NMB-R (red) and HIF-1α (green) in MDA-MB-231 cells under hypoxic or normoxic conditions.

To assess the expression of NMB-R in response to hypoxia, we analyzed the expression of the NMB-R mRNA and protein in MDA-MB-231 cells cultured in normoxic (21% O_2_) or hypoxic (1% O_2_) conditions. As shown in Figure 1A, hypoxia caused significant increases in the expression of *NMBR* mRNA. Quantitative real-time PCR demonstrated that *NMBR* mRNA expression was significantly upregulated (approximately 4.2-fold) by hypoxic treatment for 16 h (Figure 1B). We examined NMB-R levels under hypoxic conditions using western blot analysis. As shown in Figure 1C, hypoxic treatment markedly increased the levels of NMB-R in MDA-MB-231 cells. The basal and hypoxia-responsive expression of NMB-R differed among human breast cancer cell lines. The strongest induction of NMB-R by hypoxia was observed in MDA-MB-231 cells (Figure S1). Using immunocytochemistry, we confirmed the hypoxia-induced increase in the expression of NMB-R. As shown in Figure 1D, NMB-R (red) was rarely observed under normoxic conditions, and the translocation of HIF-1α (green) into the nucleus was not observed. In contrast, under hypoxic conditions, NMB-R was highly expressed in most of the cells, and nuclear translocation of HIF-1α was observed. These results indicate that NMB-R mRNA and protein expression in MDA-MB-231 cells was regulated by low oxygen tension.

### Hypoxia-induced NMBR expression is dependent on HIF-1α

**Figure 2:**
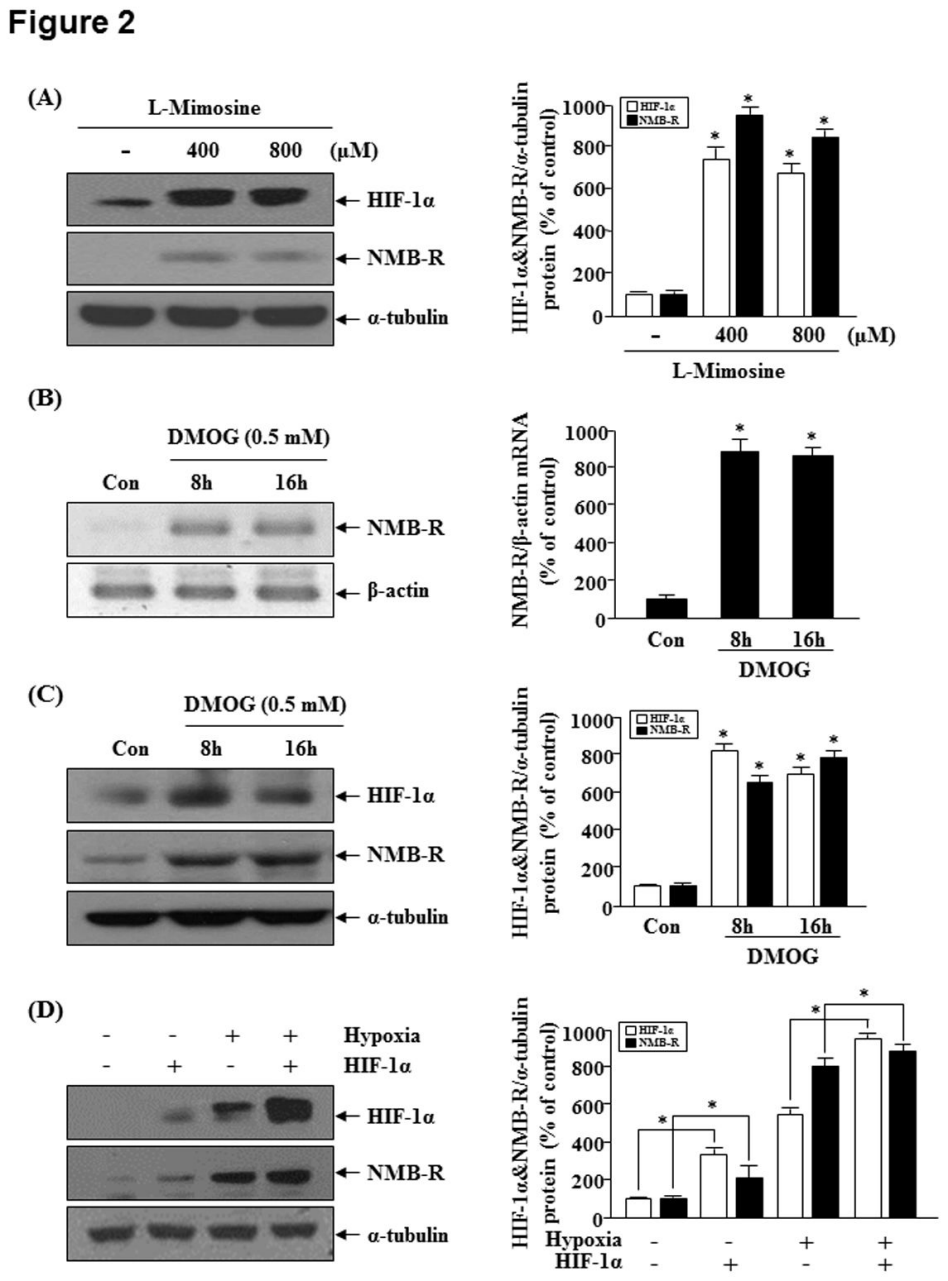
The role of HIF-1α in hypoxia-induced NMBR expression. (A) MDA-MB-231 cells were exposed to hypoxia induced using 400 or 800 μM l-mimosine for 16 h. Western blot analysis was performed using antibodies specific for human NMB-R and HIF-1α, and α-tubulin served as a loading control (left). The right panel shows the densitometric analysis assessing relative NMB-R and HIF-1α expression levels. *P<0.05 vs. control. B and C, MDA-MB-231 cells were exposed to 0.5 mM DMOG under normoxic conditions for the indicated times. (B) RT-PCR analysis was performed using specific primers for NMB-R or β-actin. The expression of NMB-R was normalized to that of the internal control β-actin (left). The density of the control bands (untreated) was defined as 100% (right). *P<0.05 vs. control. (C) Western blots were probed with anti-NMB-R or anti-HIF-1α antibodies, and α-tubulin served as a loading control (left). The right panel shows the densitometric analysis of relative NMB-R and HIF-1α expression levels. *P<0.05 vs. control. (D) MDA-MB-231 cells were transfected with an HIF-1α expression vector and then exposed to hypoxic or normoxic conditions. Western blot analysis of cell lysates using anti-NMB-R or anti-HIF-1α antibodies was performed, and α-tubulin served as a loading control (left). The graph shows the densitometric analysis of the relative NMB-R levels (right). The results represent at least 3 independent experiments. *P<0.05 vs. control empty vector.

HIF-1 is one of the master regulators that orchestrate the cellular responses to hypoxic conditions [13]. Therefore, we investigated the involvement of HIF-1 in hypoxia-induced *NMBR* expression. Small molecule inhibitors of the prolyl hydroxylase domain proteins (PHDs) block HIF-1α protein degradation and thereby activate the HIF-1 signal transduction pathway [16,17]. Therefore, we tested the effect of a hypoxia-mimetic compound, l-mimosine that inhibits PHDs. As shown in Figure 2A, NMB-R and HIF-1α expression were increased as a function of dose by l-mimosine in MDA-MB-231 cells. We tested the effects of DMOG, a cell-permeable inhibitor of HIF-α prolyl hydroxylase, on NMB-R expression in MDA-MB-231 cells. As shown in Figure 2B and 2C, treatment with DMOG led to upregulation of NMB-R mRNA and protein expression as well as HIF-1α expression even under normoxic condition. Further, we transfected MDA-MB-231 cells with a HIF-1α expression vector. As shown in Figure 2D, enforced expression of HIF-1α in normoxic or hypoxic MDA-MB-231 cells enhanced NMB-R expression compared with mock-transfected cells.

**Figure 3:**
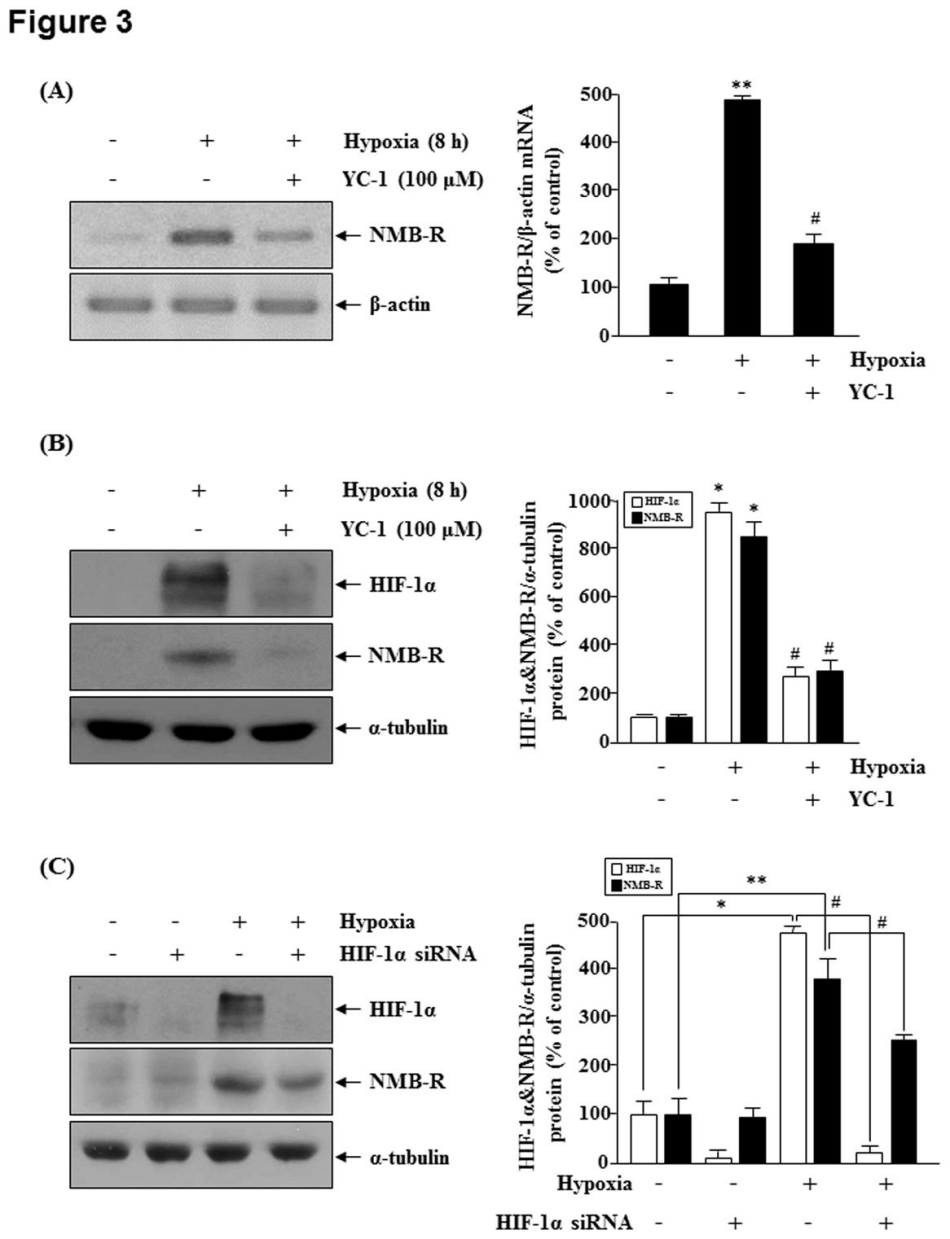
Effect of HIF-1α knockdown on hypoxia-induced NMBR expression. MDA-MB-231 cells were treated with 100 μM YC-1 under normoxic or hypoxic conditions for 8 h. (A) RT-PCR analysis was performed using specific primers for human NMB-R. β-actin served as an internal control. The graph shows the densitometric analysis of the relative NMBR mRNA levels (right). **P<0.01 vs. normoxia; ^#^P<0.05 vs. hypoxia. (B) Western blot analysis was performed using antibodies specific for human NMB-R and HIF-1α, and α-tubulin served as a loading control (left). The right panel shows the densitometric analysis of relative NMB-R and HIF-1α expression levels. *P<0.05 vs. normoxia; ^#^P<0.05 vs. hypoxia. . (C) MDA-MB-231 cells were transiently transfected with HIF-1α siRNA or control siRNAs. After transfection, the cells were incubated under normoxic or hypoxic conditions and subjected to western blot analysis to detect NMB-R or HIF-1α (left). The relative NMB-R expression level was measured in at least 3 independent experiments. *P<0.05 and **P<0.01 vs. normoxia; ^#^P<0.05 vs. control siRNA.

YC-1 reduces the hypoxia-induced accumulation of HIF-1α and the expression of HIF-1-regulated genes [18]. Therefore, we treated MDA-MB-231 cells with YC-1 under hypoxia. As shown in Figure 3A and 3B, hypoxia-mediated induction of NMB-R mRNA and protein levels were significantly reduced by YC-1. To confirm that *NMBR* was directly regulated by HIF-1α, we used a siRNA targeted to HIF-1α or a control siRNA. In the presence of HIF-1α siRNA, the level of hypoxia-induced NMB-R expression after 16 h of hypoxic exposure was markedly reduced compared with cells transfected with a control siRNA (Figure 3C). Therefore, these results indicate that HIF-1α is directly regulated by hypoxia-induced *NMBR* expression.

### HIF-1 is required for the regulation of NMBR transcription in response to hypoxia

**Figure 4:**
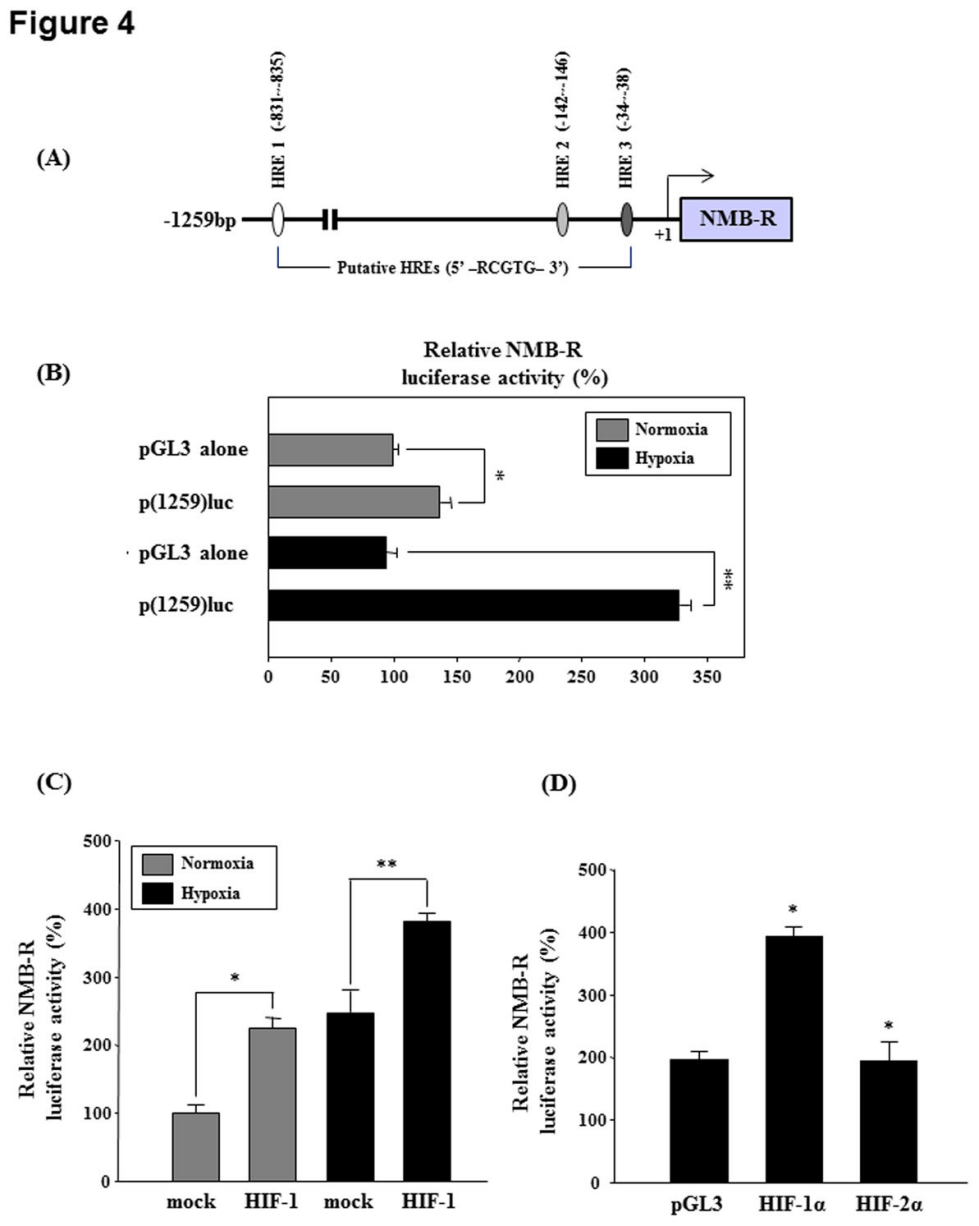
Localization of HREs in the NMBR promoter. (A) The location of putative HRE sites and the most conserved elements within the ~1 kb fragment of the 5'-flanking region of human NMBR. Putative HRE sites are defined by the core sequence 5'-RCGTG-3'. The 1259-bp fragment of the 5'-flanking region of human NMBR were subcloned upstream of a luciferase reporter gene. (B) MDA-MB-231 cells were transiently transfected with each of the NMBR reporter vectors and pCMV-β-galactosidase and then incubated under normoxic or hypoxic conditions for 24 h. The cell extracts were analyzed for luciferase activity. *P<0.05 and **P<0.01 vs. pGL3 alone. (C) MDA-MB-231 cells were cotransfected with p(1259)luc, pBOS-hHIF-1α, and pBOS-hARNT and then incubated under normoxic or hypoxic conditions for 24 h. Luciferase activity was determined. *P<0.05 and **P<0.01 vs. mock. (D) MDA-MB-231 cells were cotransfected with p(1259)luc and an HIF-1α or HIF-2α expression vector as indicated and then incubated under hypoxic conditions for 24 h. Luciferase activity was determined. Data represent 3 independent experiments. *P<0.05 vs. pGL3.

HIF-1 binds consensus hypoxia-response element (HRE) sequences in the promoters of their downstream target genes, which can induce their expression [19]. Therefore, we determined whether HREs were present in the 5´-regulatory regions of *NMBR*. As shown in Figure 4A, 3 putative HREs (HRE1, HRE2, and HRE3) were identified in the region upstream of human *NMBR* coding sequences in a cluster between −34 bp and −835 bp in the human *NMBR* promoter. Based on this information, we cloned genomic fragments containing up to 1259 bp of the 5´-flanking region of human *NMBR* and then constructed luciferase reporter vectors called p(1259)luc. The transcriptional activity of this construct was determined following transient transfection of MDA-MB-231 cells. As shown in Figure 4B, transfection with p(1259)luc resulted in a 3.3-fold increase in luciferase activity under hypoxia compared with the pGL3 empty vector. Next, to analyze the involvement of HIF-1α in activation of the NMB-R promoter, an HIF-1α expression vector was co-transfected with p(1259)luc into MDA-MB-231 cells under normoxic or hypoxic conditions. Overexpression of HIF-1α significantly increased p(1259)luc promoter activity in normoxic as well as under hypoxia (Fig. 4C). HIF-1α and HIF-2α are closely related, dimerize with a constitutively expressed β subunit (HIF-1β), and subsequently bind to the HRE in the promoters of target genes [20]. Therefore, we tested the effect of HIF-2α on hypoxia-induced *NMBR* promoter activity. As shown in Figure 4D, overexpression of HIF-2α did not affect the induction of *NMBR* promoter activity compared with cells overexpressing HIF-1α under hypoxia. These data suggest that the HIF-1 is required for hypoxic activation of *NMBR* transcription in MDA-MB-231 cells.

### Identification of a functional HRE in the NBMR promoter region

**Figure 5:**
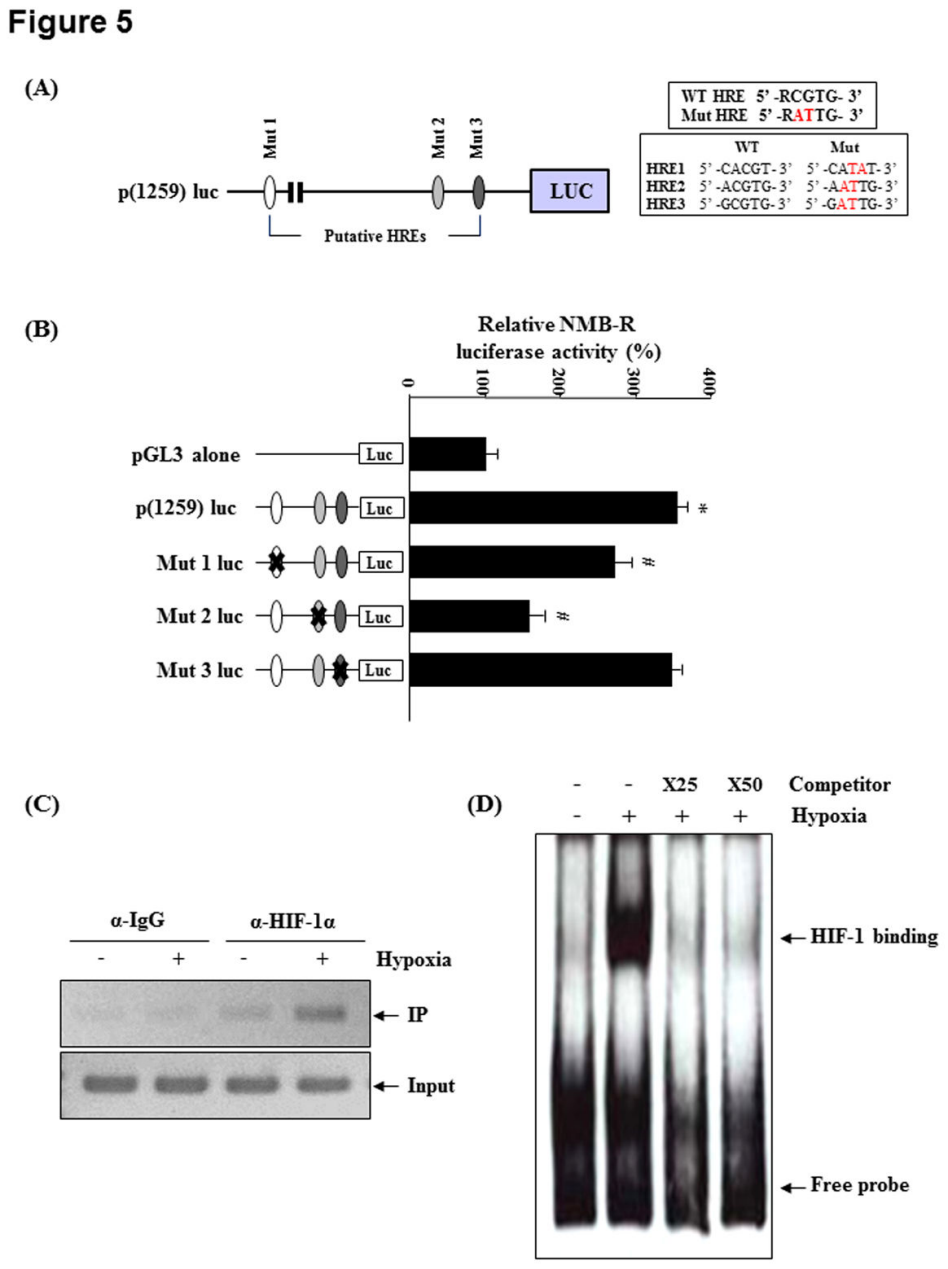
Identification of a functional HRE in the human NMBR promoter. (A) The regions of mutated HRE1, HRE2 and HRE3 (Mut 1–3) are indicated at the top. The mutations in 2 base pairs in each of the HIF-1 binding sites are indicated in the box. (B) MDA-MB-231 cells were transfected with the pGL3 basic vector, wild-type (WT), and mutant reporter vectors (Mut) and were then cultured under hypoxia for 24 h. Relative luciferase activity in cell extracts was measured using a luminometer and is expressed as relative light units. (C) MDA-MB-231 cells were cultured in atmospheres containing either 21% or 1% oxygen for 24 h. ChIP analysis was performed using control IgG or anti-HIF-1α antibodies. A sonicated cell lysate was used as an input control. (D) MDA-MB-231 cells were incubated under hypoxic conditions for 16 h. Nuclear extracts from MDA-MB-231 cells were incubated with biotin-labeled HRE-2 oligonucleotide. Arrows indicate the migration of the HRE-2 protein-DNA complex. In the competition assay, unlabeled probes in 25- and 50-fold excess were added to the reaction mixtures. Each value represents the mean of at least 3 experiments, and similar results were obtained in 3 different experiments. *P<0.05 vs. pGL3 alone; ^#^P<0.05 vs. p(1259)luc.

To determine whether a functional HRE exists within the region from −835 to +1 of the *NMBR* promoter, specific mutations were individually introduced into the core motif of the 3 putative HREs within the p(1259)luc construct (Fig. 5A). As shown in Figure 5B, although the intact p(1259)luc construct supported the hypoxic induction of luciferase activity by 3.5-fold, this induction was significantly inhibited when the HRE2 motif was mutated. However, mutation of HRE1 partially decreased hypoxia-induced p(1259)luc activation, and mutation of HRE3 did not affect promoter activity (Fig. 5B). These results indicate that the HRE2 motif (−146 to −142 bp) is essential for the hypoxic activation of the *NMBR* promoter. A ChIP assays was performed to determine whether HIF-1α was specifically recruited to these elements within the *NMBR* promoter. As shown in Figure 5C PCR products corresponding to the HRE2 region of the *NMBR* promoter were only detected under hypoxic conditions. Next, to determine whether HIF-1 can bind directly to the functional HRE2, we performed an EMSA using biotin-labeled oligonucleotide probes. As shown in Figure 5D, a clear and strong protein-probe complex was observed in nuclear extracts from hypoxia-treated MDA-MB-231 cells (lanes 2), whereas formation of this complex was inhibited by competition with an unlabeled probe (lanes 3 and 4). These results provide convincing evidence that the −146 to −142 bp sequences within the *NMBR* proximal promoter acts as a functional HRE and is capable of conferring hypoxia-mediated regulation of NMB-R expression.

### Expression of NMB-R and HIF-1α proteins in breast carcinoma

**Figure 6:**
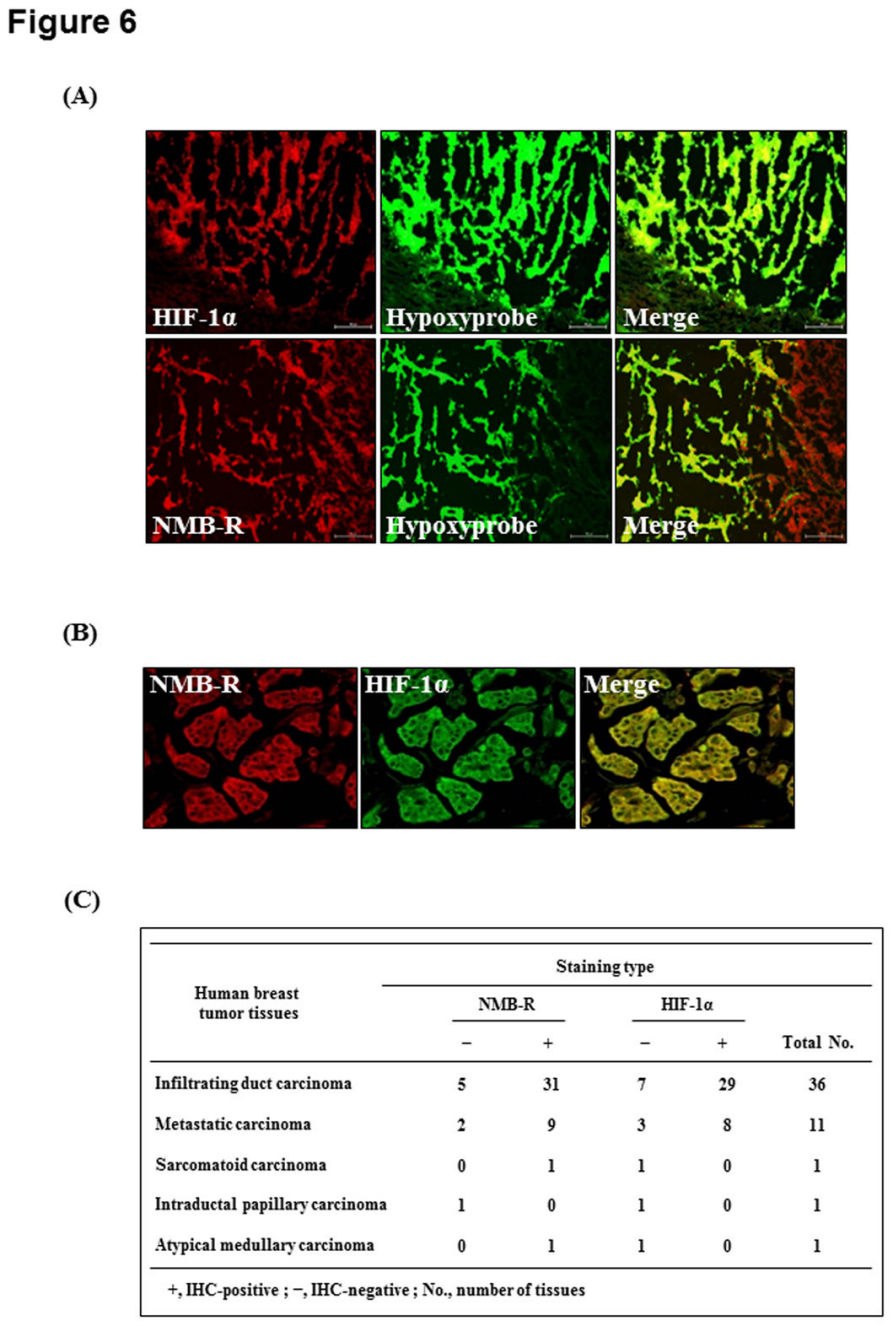
Expression of NMB-R and HIF-1α in breast carcinoma. (A) MDA-MB-231 cells were injected in the flanks of nude mice. Prior to sacrifice, MDA-MB-231 xenografts were intravenously injected with Hypoxyprobe-1 (60 mg/kg) and then embedded using OCT compound. Tissue sections from tumor xenografts were analyzed using immunohistochemistry for expression of Hypoxyprobe-1 (green), NMB-R (red), and HIF-1α (red). (B) Tissue microarray slides for human breast cancers were double-immunostained with anti-NMB-R (red) and anti-HIF-1α (green) antibodies, respectively. Sections were stained without primary anti-NMB-R or anti-HIF-1α antibodies as controls. Representative tumor sections are shown. (C) The table presents the frequency of NMB-R and HIF-1α expression in infiltrating, metastatic, sarcomatoid, intraductal papillary, and atypical medullary breast carcinoma tissues.

We investigated whether NMB-R and HIF-1α expression correlate in tumor xenografts. We first established a mouse xenograft model in which mice received subcutaneous implants of MDA-MB-231 cells and then were intravenously injected with hypoxyprobe-1 (pimonidazole hydrochloride) to visualize hypoxic regions near the center or in sporadic regions of the tumor. As shown in Figure 6A, the expression of HIF-1α was colocalized within hypoxic regions, and NMB-R was highly expresssed in pimonidazole-positive hypoxic areas. To demonstrate whether HIF-1α is associated with NMB-R expression in human neoplastic breast tissues, we performed immunohistochemical double-staining using a breast cancer tissue array. The representative immunostaining micrographs of HIF-1α and NMB-R illustrating the topological correlation are shown in Figure 6B, and relationship between HIF-1α and NMB-R expressions is summarized in Figure 6C. As shown in Figure 6C, NMB-R (42 of 50 cases, 84%) and HIF-1α (37 of 50 cases, 74%) were highly expressed in the malignant epithelia, suggesting a correlation between NMB-R and HIF-1α expression in breast tumor tissues.

## Discussion

Hypoxia commonly occurs in human solid cancers and plays an important role as a critical driving force for tumor progression that involves multiple processes such as tumor growth, angiogenesis, invasion, and metastasis [21-23]. The key regulator that mediates this process is the hypoxia-inducible heterodimeric transcription factor, HIF-1 [13]. The heterodimer comprises a constitutively expressed HIF-1β subunit and an oxygen-sensitive HIF-1α subunit, the levels of which are tightly regulated by oxygen concentration [13,19]. HIF-1α controls the transcriptional activation of a variety of genes in the divergent signaling pathways involved in cell proliferation, survival, energy metabolism as well as tumor metastasis and angiogenesis [23,24].

Several lines of evidence indicate that mammalian bombesin-like peptide receptors, including the GRP and NMB receptors, are frequently overexpressed by a variety of tumor cell lines and tumor specimens from patients with lung, colorectal, gastric, prostate, and breast cancers [8,10,25-27]. Overexpression of bombesin-like peptide receptors promotes tumor development and progression by stimulating cancer cell proliferation and migration [28-30]. Recently, targeting GRP receptor (GRP-R) represents a useful therapeutic strategy to treat some human malignancies [31,32]. GRP-R antagonists, antisense oligonucelotides for the GRP-R, monoclonal antibodies against GRP, and drug-linked or radiolabeled GRP analogs showed effective anticancer activity in experimental therapy and clinical applications [32-35]. We previously reported that inhibition of NMB-R signaling using an NMB-R antagonist reduces the *in vivo* and *in vitro* tumor growth of breast cancer cells by inducing cell-cycle arrest and apoptosis [8]. Also, NMB-R antagonist inhibits the proliferation of C6 glioma cells and regulates intracellular signaling in lung cancer cells [29,36]. Thus, NMB-R may be an attractive target for diagnosis and treatment of different types of cancers overexpressing NMB-R. These facts highlight the need for further investigation in regards to cancer-specific strategies.

Human *GRPR* is upregulated by the recruitment of the transcription factor CRE-binding protein (CREB) to a cAMP-response element (CRE)-binding site within the *GRPR* promoter in cancer cells [37,38]. Here, we provide the first insights regarding the molecular mechanisms of *NMBR* regulation in breast cancer cells by demonstrating that hypoxia increased NMB-R expression in human cancer cells and that HIF-1α directly bound to and transactivated the *NMBR* promoter in response to hypoxia. HIF prolyl hydroxylases (PHDs), PHD-1, -2 and -3, mediate oxygen-dependent degradation of HIF-1α subunit [16,17]. Considering the differences of enzyme activity of PHDs and tissue distribution of PHDs may result in a graded or cell- or tissue-specific response to hypoxia [16,17], it would be of interest to explore the expression of cancer type-specific *NMBR* and its regulation in human cancers.

Ectopic expression of NMB-R stimulates the mitogenic responses of Rat-1 fibroblasts by promoting DNA synthesis and cell proliferation [39]. To determine whether upregulation of NMB-R alters the biological properties of MDA-MB-231 breast cancer cells under hypoxic conditions, we assessed the effect of on their proliferation by NMB-R overexpression or knockdown. The results revealed that NMB-R overexpression promotes breast cancer cells proliferation under hypoxia, while NMB-R knockdown significantly decreases proliferation of hypoxic tumor cells (Fig. S2). Accumulating evidence reveals that NMB acts through the NMB-R to promote the proliferation of various types of normal and cancer cells through activation of ERK1/2 signaling [6,29,40,41]. ERK1/2 activated by hypoxia induces HIF-1α expression through the generation of reactive oxygen species and activates Rac1 in breast cancer cells [42]. We are currently investigating the molecular mechanism(s) underlying NMB-R-mediated proliferation of hypoxic cancer cells.

In conclusion, the present study reveals the molecular mechanisms involved in regulating the expression of *NMBR* as a novel hypoxia-inducible gene. Moreover, we show that upregulation of NMB-R by hypoxia is mediated by HIF-1α-dependent transcriptional activity in breast cancer cells. Our findings may provide new insights into the role of NMB-R in tumor progression and may lead to the development of novel therapeutic approaches to target cancer cells.

## Supporting Information

Figure S1 **Effect of hypoxia on NMBR expression in human breast cancer cell lines.** (A) MDA-MB-231, MDA-MB-468, T47D and MCF-7 cells were incubated under normoxic (21% O_2_) or hypoxic (1% O_2_) conditions for 16 h. The expression of NMB-R was assessed using western blotting and antibodies specific for human NMB-R. β-actin was used as the loading control. (B) The graph shows the densitometric analysis of the relative NMBR mRNA levels. The density of the control bands (normoxia) was defined as 100%. The results represent at least 3 independent experiments. *P<0.05 vs. normoxia.
(TIF)

Figure S2 **Functional role NMB-R in MDA-MB-231 cell proliferation under hypoxia.** MDA-MB-231 cells were transfected with empty vector, NMB-R expression vector, control siRNA or NMB-R siRNA and then exposed to hypoxic conditions for the indicated times. Analysis of BrdU incorporation and total DNA content in a proliferating MDA-MB-231 cell line using the BrdU Flow kit. DNA synthesis rates were measured by determining the percentage of BrdU-positive cells using FACS analysis. The results shown are representative of at least three independent experiments.
(TIF)
